# End-Point Affinity Estimation of Galectin Ligands by Classical and Semiempirical Quantum Mechanical Potentials

**DOI:** 10.1021/acs.jcim.4c01659

**Published:** 2025-01-04

**Authors:** Jan Choutka, Jakub Kaminský, Ercheng Wang, Kamil Parkan, Radek Pohl

**Affiliations:** † Institute of Organic Chemistry and Biochemistry of the Czech Academy of Sciences, Gilead Sciences & IOCB Research Centre, Flemingovo nám. 2, 166 10 Prague, Czech Republic; ‡ Zhejiang Laboratory, Hangzhou 311100, China; § Department of Chemistry of Natural Compounds, University of Chemistry and Technology Prague, Technická 5, 166 28 Prague, Czech Republic

## Abstract

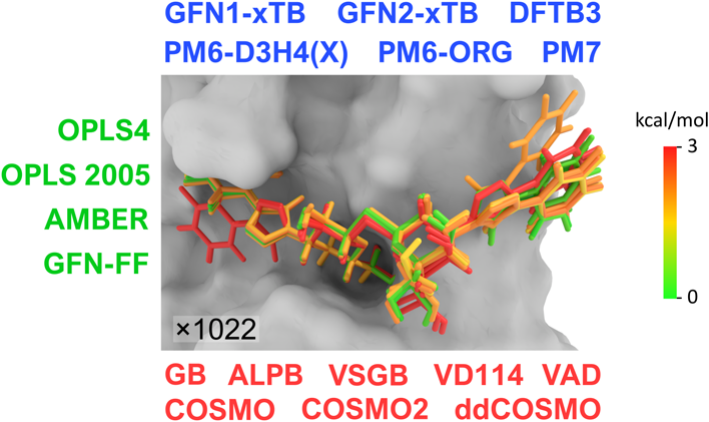

The use of quantum mechanical potentials in protein–ligand affinity prediction is becoming increasingly feasible with growing computational power. To move forward, validation of such potentials on real-world challenges is necessary. To this end, we have collated an extensive set of over a thousand galectin inhibitors with known affinities and docked them into galectin-3. The docked poses were then used to systematically evaluate several modern force fields and semiempirical quantum mechanical (SQM) methods up to the tight-binding level under consistent computational workflow. Implicit solvation models available with the tested methods were used to simulate solvation effects. Overall, the best methods in this study achieved a Pearson correlation of 0.7–0.8 between the computed and experimental affinities. There were differences between the tested methods in their ability to rank ligands across the entire ligand set as well as within subsets of structurally similar ligands. A major discrepancy was observed for a subset of ligands that bind to the protein via a halogen bond, which was clearly challenging for all the tested methods. The inclusion of an entropic term calculated by the rigid-rotor-harmonic-oscillator approximation at SQM level slightly worsened correlation with experiment but brought the calculated affinities closer to experimental values. We also found that the success of the prediction strongly depended on the solvation model. Furthermore, we provide an in-depth analysis of the individual energy terms and their effect on the overall prediction accuracy.

## Introduction

Predicting protein–ligand affinities is one of the pivotal tasks in rational drug design. To this day, accomplishing this task by computational methods, *prospectively and with sufficient accuracy*, has proven to be difficult, but possible in principle. In general, predicting affinity requires computing the free energy change associated with protein–ligand complex formation, ideally including both enthalpic and entropic contributions.

One possible approach to this problem is to extract the free energy change from a molecular dynamics simulation by means of statistical mechanics. The most established methods in this group are thermodynamic integration (TI) or free energy perturbation (FEP), among other variants.^1,2^ These methods often use unnatural (“alchemical”) transitions between two states—either two chemical species or two environments. In the context of protein–ligand binding, this methodology is most commonly used to estimate the relative affinity between two (or more) ligands by alchemically morphing one ligand into another through several intermediate simulations. While this approach is theoretically rigorous, it is limited to networks of similar ligands, precluding the comparison between ligands with marked structural differences. Furthermore, the computational cost of such methods has so far limited their large-scale use and the deployment of more expensive potentials beyond classical force fields.

One way to circumvent the above limitations is to simplify the problem of protein–ligand affinity prediction by only considering the thermodynamic end points (end states) of the binding process.^3^ This so-called end-point approach has the inherent advantage of lower computational cost because only the thermodynamic states of interest, but not the path between them, need to be evaluated. In turn, this simplification allows the use of a higher level of theory such as semiempirical quantum mechanical (SQM) or possibly even fully quantum mechanical (QM) potentials. Another intrinsic advantage of the end-point approach is, at least in principle, the ability to efficiently compare ligands from different regions of chemical space and score them consistently against each other.

Among the higher-level methods that could be used for end-point scoring of protein–ligand binding affinities, modern SQM methods^4,5^ stand out as a promising compromise that offers an increased theoretical level compared to classical force fields, while maintaining reasonable computational cost. Among the most successful SQM methods are those based on the NDDO (neglect of diatomic differential overlap) approximation to the Hatree–Fock formalism. Out of these, the PM6 method was the first that was parametrized with organic systems in mind.^6^ Later, several versions of corrections for noncovalent interactions were introduced to PM6 with the aim of improving the performance on protein–ligand systems. These corrections include dispersion (D), hydrogen bonding (H), and halogen bonding (X) terms, and the D3H4X version^7−9^ is the most recent. A standalone reparametrization of the PM6 method focusing on protein–ligand systems was also recently introduced and termed PM6-ORG.^10^ Currently, the newest member of the PMx group is the PM7 method, which includes dispersion and hydrogen bonding terms by default.^11^

Another group of SQM methods is based on the simplification of the Kohn–Sham density functional theory, as opposed to Hartree–Fock. Among these, density functional tight-binding (DFTB)^12−14^ is the most established. This method is currently available up to third-order expansion (DFTB3) and is typically used with a self-consistent redistribution of charges (SCC). To account for missing dispersion, DFTB is usually complemented by D3 or D4 corrections.^15,16^ Additionally, a hydrogen bonding correction was introduced by a D3H5 scheme in a self-consistent manner.^17^ More recently, a family of extended tight-binding (xTB) methods was established as an alternative to DFTB.^18^ While the initial method of this family, GFN1,^19^ included explicit hydrogen and halogen bonding corrections, the more recent GFN2 method^20^ needs no such corrections and treats all noncovalent interactions internally. This method also features the D4 dispersion term and multipole electrostatics by default.

A key aspect of protein–ligand affinity predictions is the treatment of solvation effects. In contrast to molecular dynamics-based methods, where the system can be continuously sampled against explicit solvent molecules, solvation effects in end-point methods are typically treated in a mean-field way. This is accomplished by using implicit solvation models, in which the solvent is simulated as a dielectric continuum. The most established continuum models used in protein–ligand modeling are those based on the generalized Born (GB) approximation, or numerical solutions to the Poisson–Boltzmann (PB) equation.^21,22^ Polarized continuum models, especially the conductor-like screening model (COSMO) in its various parametrizations, have also seen a lot of use.^23^ The electrostatic solvation energy obtained from these continuum models has frequently been complemented with an additional term based on solvent-accessible surface area (SASA), which aims to approximate the nonpolar contribution to the solvation energy. This term is usually constructed by scaling SASA with a surface tension coefficient and sometimes further augmented by a solute–solvent attractive term.^24−26^

The task of scoring protein–ligand affinity within the framework of the end-point approach can be divided into two consequent problems. First, the correct pose of the ligand in the binding site of the receptor must be determined, and second, the free energy difference between the associated and dissociated end points for that given pose must be determined. In a prospective situation where the binding pose is not known, it must be found by a suitable sampling step–usually a docking algorithm. The docked pose is then typically optimized to the nearest energy minimum, or alternatively it could be sampled by molecular dynamics to produce an ensemble of structures. Once a candidate conformation of the complex is determined, it can be scored by evaluating the potential energy of the critical end points in the thermodynamic cycle of protein–ligand binding.

To date, many studies have investigated molecular mechanics methods with implicit solvation, collectively termed MM/GB(PB)SA, on a plethora of systems with variable success.^27−29^ Several groups have also tested higher-level methods, mostly SQM, typically on small sets of ligands. To name some, Merz and co-workers developed a scoring framework^30−33^ based on AM1 and PM3 methods with PB solvation and applied it on a range of protein targets.^34^ Ryde and co-workers tested AM1, RM1, and PM6 methods coupled with DH2 corrections and COSMO solvation on three protein targets with less than ten ligands for each target.^35^ More work using the PM6 method with D, H, and X corrections was done by Hobza and Řezáč.^36−40^ Recently, their efforts culminated in the development of the SQM2.20 scoring function based on the PM6-D3H4 method and an updated COSMO-based solvation model.^41^ This scoring function was tested on a diverse benchmark set of ten protein–ligand systems, with up to a few tens of ligands for each target.^42^ Grimme and co-workers described a QM-level scoring methodology based on HF-3c and PBEh-3c composite methods combined with the COSMO-RS solvation model and tested it on two protein targets, also with a few tens of ligands for each.^43^ The various efforts to include QM methods into the framework of end-point scoring of protein–ligand complexes have been reviewed elsewhere and we refer the reader there for more comprehensive picture.^44^

Following up on the above-mentioned efforts, we believe that a comprehensive evaluation of SQM methods, including the most modern ones, on a larger real-world set of ligands is desirable. It is also important to determine whether the increased computational cost of the SQM methods can be justified by increased accuracy over the cheaper force field level.

In this study, we assembled a large set of small compounds with known affinities toward Galectin-3 and -1 (Gal-3 and -1), which are sugar-binding proteins that play a role in various pathological conditions including inflammation, cancer, pulmonary fibrosis, or viral entry.^45,46^ The resulting ligand set contains more than a thousand ligands with *K*_d_ values obtained from fluorescence anisotropy assay and thus represents the largest set of galectin inhibitors usable for in silico evaluations and one of the largest curated ligand sets against a single target. We then docked this set into a crystal-derived conformation of Gal-3, and consequently used the docked poses for end-point scoring with various methods under consistent computational workflow. Our main aim was to evaluate the ability of several force fields and SQM methods in combination with available implicit solvation models to estimate the binding enthalpies and binding free energies of the ligands to Gal-3 and compare them with experimental binding free energies. The results provide insights into the performance of the tested methods under conditions close to those that would be used in a prospective scenario.

## Methods

### Overall Workflow

**Figure 1:**
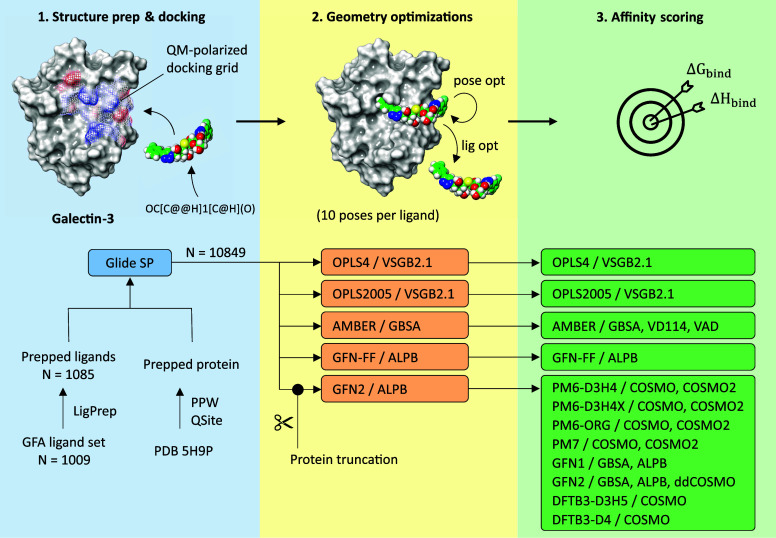
Computational workflow for QM-polarized docking and consequent end-point estimation of galectin-ligand affinities.

The overall workflow employed in this study is depicted in Figure 1. We relied on molecular docking to sample several candidate poses of a ligand in the binding pocket of Gal-3, and then scored these poses by higher-level methods using an end-point thermodynamic cycle. First, ionization and tautomeric states of the ligands were generated to capture their realistic states in solution. These ligand states were then docked into the canonical binding pocket of Gal-3, which was characterized with partial charges obtained at the DFT level. The 5H9P PDB structure of Gal-3 was used, which contains subsites C and D in a conformation that accepts the native disaccharide motif and also harbors a cryptic opening of Arg144 that accepts C-3 aromatic decorations present in many ligands in the GFA ligand set. For each ligand state, up to ten poses (exactly ten for all ligands except one) were generated. This means that for a ligand with only one ionization and tautomeric state, ten poses were generated, for a ligand with two states, 20 poses were generated, and so on. Overall, from 1009 ligands from the GFA ligand set with known *K*_*d*_ values for Gal-3, 1085 states and 10,849 docked poses were generated.

The docked poses were then optimized by constrained optimizations, where only the ligand was allowed to move, and the protein was kept frozen. The motivation behind keeping the protein frozen is 2-fold. First, optimization of the docked poses with a flexible receptor and subsequent scoring of multiple free receptor end points adds a significant computational cost. And second, modeling flexible binding pockets such as that of Gal-3 by simple minimization does not sufficiently capture the conformational dynamics of the protein. An ensemble of structures would need to be sampled to properly model the flexibility of the protein.

After the pose optimization, the structure of the ligand from the optimized complex was taken and optimized alone into the nearest minimum to obtain the ligand conformational strain. These geometric operations were performed at several different levels. At the force field level, separate geometries were generated by each force field, which were then used for affinity scoring at the respective levels. For modeling at SQM level, the GFN2/ALPB method was used to generate the geometries. For all modeling at the SQM level, the protein was truncated to a globule defined as residues within a 12 Å distance from the ligand in 5H9P structure (1152 atoms). Dangling valences were capped with hydrogens. The GFN2-optimized geometries were then used for single-point scoring with all the other SQM methods used in this study. For calculating the entropic contributions ( terms), the receptor was further truncated to an even smaller 6 Å globule (417 atoms).

**Figure 2:**
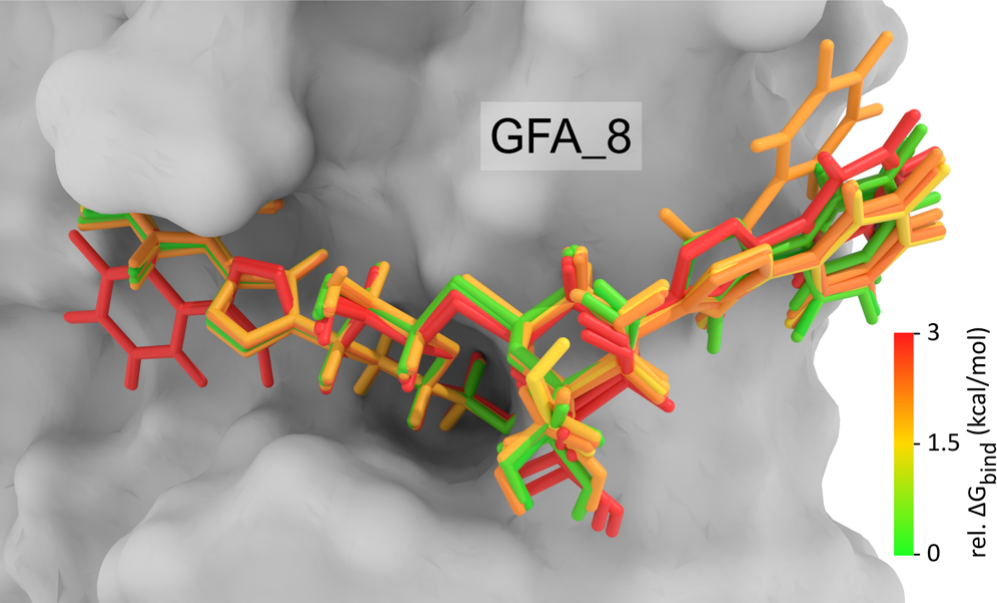
Ensemble of poses generated by docking and pose optimization at the SQM level for compound GFA_8. Poses are colored according to the relative Δ*G*_bind_ calculated by the DFTB3-D4/COSMO method.

For ligands where the *K*_d_ value was reported as *lower than* or *higher than*, the given value was treated as the actual *K*_d_ value. The final binding enthalpy and binding free energy scores for a given ligand were determined as the minimal value from all evaluated states and poses of the given ligand. An example of a pose ensemble of compound GFA_8 (GB0139) generated by our sampling procedure and rescored at the SQM level is shown in Figure 2. The lowest energy pose (green) was used for further analysis.

### Protein and Ligand Structure Preparation

Preparation of the protein and ligands, as well as docking, were carried out in the Schrödinger suite^47^ (version 2023-3). The PDB structure 5H9P (carbohydrate recognition domain of Gal-3 with GB0139 ligand) was used for all the work in this study. The structure was prepared by Protein Preparation Workflow,^48^ in which bond orders were assigned, hydrogens were added, ionization states were generated by Epik^49^ at pH = 7.4 ± 2.0, and H-bonds were optimized by PROPKA. All water molecules were erased and the structure was relaxed by restrained minimization. The atomic electrostatic potential (ESP) charges of the prepared 5H9P complex were calculated by a single-point QM/MM calculation in QSite,^50,51^ where the ligand and binding site residues (namely side chains from residues Arg144, Asp148, His158, Asn160, Arg162, Glu165, Asn166, Asn174, Trp181, Glu184, Arg186, and Ser237) were treated at the B3LYP/6-31G** level, while the rest of the protein was treated using the OPLS2005 force field.^52,53^ The junctions between the side chains modeled by QM and the rest of the protein were treated by the frozen orbital method.^50,51^

Ligands were converted from SMILES strings to 3D models using LigPrep with the OPLS4 force field.^54^ Tautomers and ionization states were generated using Epik Classic at pH = 7.0 ± 2.0. Out of 1009 compounds with known *K*_d_ for Gal-3, 1085 structures were generated by LigPrep (ionization and tautomeric states).

### Docking

Two docking grids were generated—a regular-sized grid set up for a ligand length of 25 Å (inner box 10 Å, outer box 35 Å), and a larger grid set up for a ligand length of 35 Å (inner box 10 Å, outer box 45 Å). In both cases, the box was centered on the ligand centroid in the 5H9P structure. When generating the docking grid, the ESP charges from the previous QM/MM calculation were assigned to the binding pocket of the receptor, while the rest of the receptor was assigned with OPLS 2005 charges. The hydroxyl from Ser237 was treated as a rotatable group. Docking was performed in Glide^55^ using the SP procedure (GlideScore version SP5.0) with flexible ligand sampling. To soften the close contacts during docking, the VdW radii of the ligand nonpolar atoms (partial charge less than 0.15) were scaled by a factor of 0.8. Partial charges for ligands were taken from OPLS4. Epik state penalties were added to the docking score. Selenium was additionally defined in Glide as a generic nonpolar sp3-hybridized atom. In the conformer generation step (ConfGen module), enhanced sampling (2×) was used. Ligands with molecular weight < 800 g/mol were docked to the regular-sized box, while the larger box was used for ligands with molecular weight > 800 g/mol (8 ligands). For ligands with *K*_d_ > 5 μM, the number of poses passed through the initial Glide screen was raised from the default 5000 to 50,000 per ligand to achieve more exhaustive pose sampling. Postdocking minimization was performed on 20 best poses per ligand, and the best 10 poses at most were written out as a docking output for further processing. In total, 10849 docked poses were generated by docking.

### Pose Optimizations and Single-Point Energy Scoring

#### Schrödinger Prime

Calculations with OPLS4^54^ and OPLS2005^52,53^ force fields in combination with the VSGB2.1 solvation model^56^ were carried out within the MM/GBSA module in Schrödinger Prime (v3.0), Schrödinger suite^47^ (version 2023-3). Pose optimizations were performed using the “minimize” option, with no flexible residues.

#### AMBER

All modeling was performed in AMBER18.^57^ The proteins were parametrized using the AMBER ff03 force field, and the ligands were parametrized using the general AMBER force field (gaff v1.81) complemented by the AM1-BCC charge model. The GB5 variant (igb = 5) of GBSA was used (mbondi2 atomic radii, OBCII). During pose optimizations, a strong constraint of 100 kcal/mol/Å^2^ was applied. The optimization of free ligands was done without any constraints. Both minimizations comprised 1000 cycles of the steepest descent method followed by the conjugate gradient method. The pmemd.MPI engine from the AMBER18 package was utilized to execute these minimization steps. The solvation models using variable dielectric regimes VD114-GBSA^58^ and VAD-GBSA^59^ were modeled using a modified version of the MMPBSA.py script^60^ as described^58^ previously.

#### Extended Tight-Binding (xTB)

The xTB program^18^ (version 6.6.1) was used for calculations with GFN-FF,^61^ GFN1,^19^ and GFN2^20^ methods in combination with GBSA and ALPB^62^ implicit solvation models. For pose optimizations at the GFN2/ALPB level, the protein globule was constrained using exact fixing (for GFN2) or constraining potential (for GFN-FF). The default level of accuracy (--opt normal) was used for both complexes and free ligands. The  terms were calculated with the GFN2 method in the gas phase using the biased Hessian (single-point Hessian)^63^ approach (--bhess). The solvation model that we denote here as ddCOSMO refers to the SCF part of the CPCM-X model,^64^ without the post-SCF contribution, and was calculated in the 6.7.0 version of xTB. We were not able to include the full CPCM-X solvation energies because the post-SCF part of this calculation failed to converge on many structures in our data set.

#### Density Functional Tight-Binding (DFTB+)

The binding enthalpy of the GFN2-optimized poses was also evaluated by single-point calculations at the DFTB level using Self-Consistent redistribution of Charges (SCC)^65^ in the DFTB+ program (version 23.1).^66^ The DFTB3 modification^67^ of the DFTB-SCC method supplemented with the D3^15^ (within the D3H5 variant with additional corrections for noncovalent interactions^17^) or D4^16,68^ dispersion correction was used. The DFTB Hamiltonian was constructed using tabulated integrals and parameters derived by Third-Order Parametrization for Organic and Biological Systems (3OB), as given in the 3ob-3-1 Slater-Koster files.^69−71^ For modeling in DFTB+, the ligand GFA_416 (selenodigalactoside) was omitted because the 3ob-3-1 Slater-Koster files used here do not contain parameters for selenium. The effect of water was approximated using the conductor-like screening model (COSMO),^72^ as implemented in DFTB+.

#### Molecular Orbital Package (MOPAC)

For single-point calculations with the PM6-D3H4,^7^ PM6-D3H4X,^8^ PM6-ORG,^10^ and PM7^11^ methods in conjunction with COSMO^72^ or COSMO2^41^ solvation models, the open-source implementation of MOPAC^73^ (version 22.1.1) was used. In MOPAC, the localized molecular orbitals algorithm (MOZYME) was used to solve the SCF equations. The molecular mechanics correction (MMOK) was used, and the dielectric constant for COSMO and COSMO2 was set to 78.4 (EPS = 78.4). When using the COSMO2 solvation model, the reoptimized atomic radii were used, and the nonpolar solvation term was added by scaling the COSMO area by effective surface tension parameter (ξ = 0.046 for PM6 and ξ = 0.042 for PM7), as reported by Řezáč et al.^41^

### Affinity Scoring

To estimate the affinity of ligands to Gal-3, we have used physics-based partitioning into several energy terms to describe the most important contributions to receptor–ligand binding. We use two quantities to estimate affinity: binding enthalpy Δ*H*_bind_ and binding free energy Δ*G*_bind_. These quantities are calculated by the following equations:
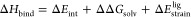
1
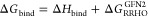
2

The term Δ*E*_int_ corresponds to the gas-phase interaction energy and is obtained by single-point energy calculations as the difference between associated receptor–ligand complex (*E*_gas_^com^) and the dissociated receptor (*E*_gas_^rec^) and ligand (*E*_gas_^lig^) structures in the same conformations as in the complex:
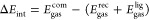
3

The other major term is the binding desolvation energy ΔΔ*G*_solv_, which is calculated using the same geometries as the interaction energy. This term corresponds to the cost of removing solvent from the areas of contact between the receptor and ligand and is calculated as the difference between solvation energies of the complex (Δ*G*_solv_^com^) and dissociated receptor (Δ*G*_solv_^rec^) and ligand (Δ*G*_solv_^lig^) structures:
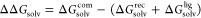
4

The solvation energies Δ*G*_solv_ are calculated using implicit solvation as the difference between the structures in the gas phase and in solvent. The terms Δ*E*_int_ and ΔΔ*G*_solv_ oppose each other, with the former being favorable, and the latter unfavorable. Hence, a delicate compensation between the computed values of Δ*E*_int_ and ΔΔ*G*_solv_ is important.

 corresponds to the conformational strain of the ligand. This term captures the cost of straining the ligand into bound conformation from its relaxed state. We calculate  by taking the ligand conformation from the complex and minimizing it to the nearest energy minimum. The sums of the conformational and solvation energies of these two conformations are then subtracted as follows:
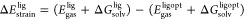
5

Herein, the terms *E*_gas_^lig^ and Δ*G*_solv_^lig^ describe the ligand in the bound conformation, and the terms *E*_gas_^ligopt^ and Δ*G*_solv_^ligopt^ describe the ligand in the nearest minimum. As calculated here,  captures only the local conformational penalty, but neglects a possible global penalty arising from the energy difference between the relaxed conformation nearest to the bound state and a conformation in the global minimum, which may or may not be the same. Furthermore, the conformational strain of the receptor () should in theory also be included as an important enthalpic contribution to binding. However, this term was neglected in this study, as we kept the Gal-3 structure frozen into a single conformation at all times, and thus  equals zero.

Lastly,  describes the loss of the translational, rotational and vibrational entropy upon binding. We calculated this term by harmonic frequency analysis using Grimme’s modification of the rigid-rotor-harmonic-oscillator (RRHO) approximation, where low frequencies are treated as rigid rotors.^74^ To limit the errors arising from imaginary frequencies obtained from nonequilibrium structures, the frequencies were calculated using the biased Hessian approach.^63^ is calculated as the difference in RRHO terms between the complex () and the sum of the free receptor and free ligand () as follows:
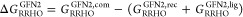
6

The individual RRHO terms are calculated at 298.15 K and include zero-point-vibrational energy and thermostatistical corrections. In contrast to all other energy terms in our scoring scheme, which were computed separately by each tested method, the  term was calculated only by the GFN2 method in the gas phase and added to the Δ*H*_bind_ score from all tested methods in this study.

## Results and Discussion

### GFA Ligand Set

To obtain a sample for our investigation, we have searched the literature and collated a set of ligands with known dissociation constant (*K*_d_) for Gal-1 and/or Gal-3. Collating sets of affinity values from various literature sources and different assays can lead to large uncertainties due to assay specifics and experimental inconsistencies. It was shown that Kendall‘s correlation coefficient between IC_50_ values obtained from different assays in the ChEMBL database can be as low as 0.51.^75^ More alarmingly, the values of *K*_d_ (*K*_i_) from different assays were shown to have similarly poor correlation, pointing to major inconsistencies in existing experimental data. To limit the uncertainty in our ligand set, we have only included ligands with reported *K*_d_ values obtained from a single assay, specifically the fluorescence anisotropy (fluorescence polarization) assay. The fluorescence anisotropy assay has been widely used for galectin inhibitors, resulting in abundant data throughout the literature and patents. When curating our set, galectin ligands with *K*_*d*_ values reported from other assays, and ligands with reported IC_50_ values were not included. To compare the experimental affinities with the computed ones, the *K*_d_ values from the literature were converted to experimental binding free energies Δ*G*_bind_^exp^ by
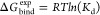
7

The resulting Galectin Fluorescence Anisotropy (GFA) ligand set includes 1022 unique compounds, of which 635 have reported *K*_d_ value against Gal-1 and 1009 against Gal-3. For a few commonly evaluated ligands, more than one *K*_*d*_ value was found, and thus the set contains a total of 1034 data points (645 *K*_d_ values for Gal-1 and 1021 for Gal-3). The ligand set was gathered from 46 sources (27 papers, 18 patents, and 1 dissertation) and is up-to-date as of 2023. The GFA ligand set is categorized into eight ligand series, which were grouped based on ligand structural motifs (see Table 1). We also provide categorization according to the number of sugar rings in the core of the ligand (820 are *monosaccharide*-based, 194 are *disaccharide*-based, and 8 are characterized as *other*). Furthermore, we have curated the set to include information about the putative halogen bonding with a glycine residue (Gly182 in Gal-3, Gly69 in Gal-1).^76^ The putative halogen bond is expected for 471 ligands and occurs in the series of *(Het)aryl-C-galactosides* and *Alpha-thiogalactosides*. All ligands in the set are further characterized by their molecular weight, topological polar surface area, number of rotatable bonds, and number of hydrogen bond acceptors and donors. See Supporting Information (Figures S1–S3) for graphical representations of the distribution of affinities and physicochemical properties. The GFA set is provided in the form of SMILES strings and is made available together with this manuscript (see Associated Content).

**Table 1:** Composition of the GFA Ligand Set

		Galectin-1		Galectin-3	
series	# of unique compounds	# of affinities	*K*_d_ range (μM)	# of affinities	*K*_d_ range (μM)
(Het)aryl-*C*-galactosides	188	130	0.017 to 2100	188	0.002 to >3000
Alpha-thiogalactosides	465	275	0.006 to >100	465	0.002 to 63
Beta-thiogalactosides	59	24	100 to >2000	59	1.7 to 5900
LacNAc derivatives	81	29	6.7 to 280	83	0.014 to 160
Lactose derivatives	11	12	4.4 to 500	13	2.2 to 231
Monosaccharide derivatives	108	87	23 to >10,000	97	0.54 to 10,000
Not a sugar	1	1	>500	1	0.15
Thiodisaccharide derivatives	109	87	0.003 to 200	115	0.0003 to 4300
**total**	**1022**	**645**	**0.003 to >10,000**	**1021**	**0.0003 to 10,000**

### Docking Results

**Figure 3:**
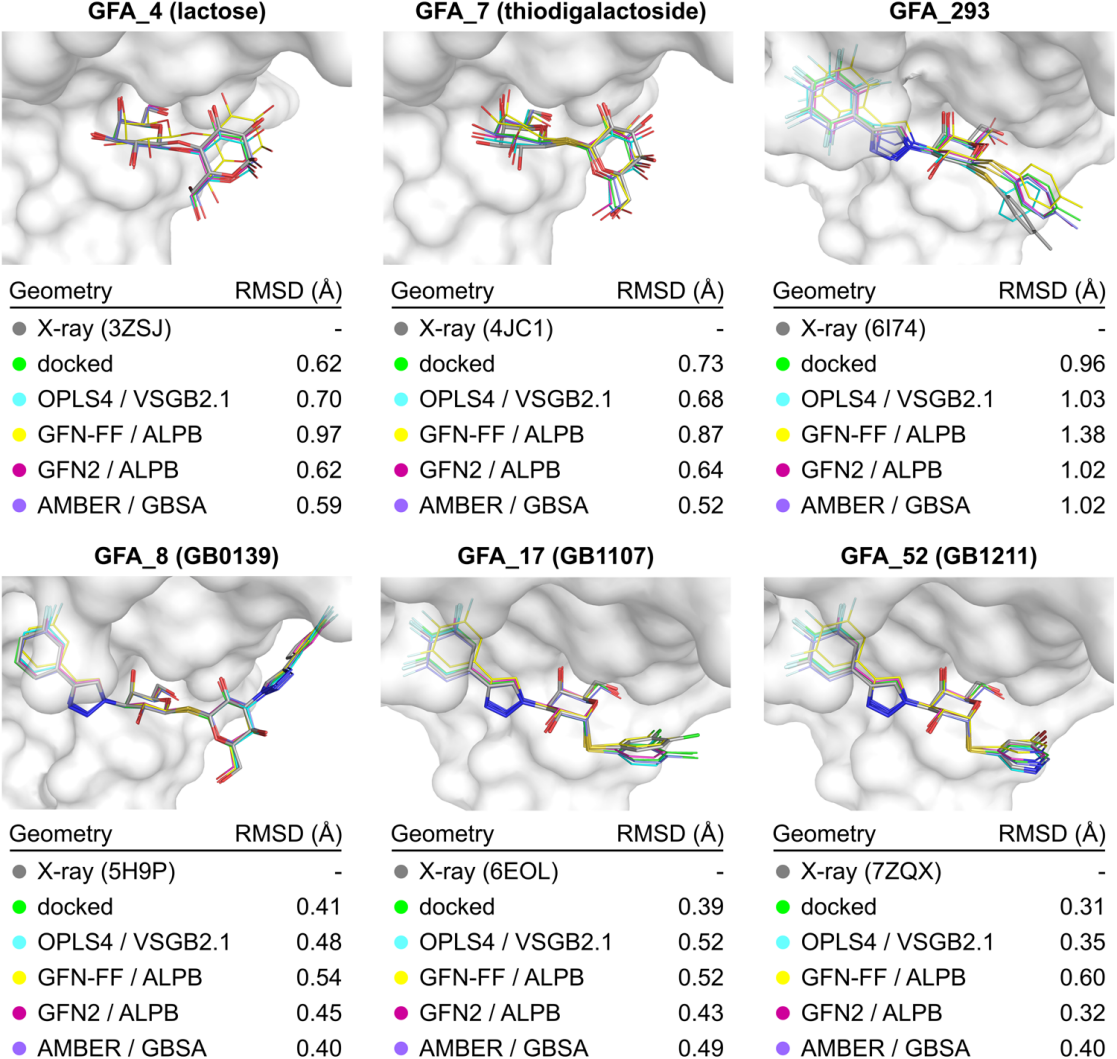
Quality assessment of the modeled poses by comparison with known X-ray structures for selected ligands. Overlays were obtained by aligning the protein backbone of the modeled complex (using protein conformation from 5H9P) with the X-ray conformation of a complex of Gal-3 with the given ligand. Heavy-atom RMSD between ligand conformations from model and from X-ray was used.

We have evaluated the quality of the docked poses of six ligands for which crystal geometries are available. Figure 3 shows overlays of crystal structures (gray) and ligand geometries obtained by docking (green) and pose optimizations (other colors). Shown are the poses with the lowest RMSD to the crystal pose for each method. All in all, we found that for the six evaluated ligands, the docked poses are in very close agreement with the crystal geometries (RMSD < 1 Å). We also found by random inspection that our docking protocol generally gives reasonable poses where the β-d-galactose moiety is consistently being docked into the canonical binding pocket in at least one pose. Thus, we expect that the docking accuracy measured for the selected six compounds is representative of the whole ligand set, although we were not able to systematically assess this due to a lack of crystal structures. Among the optimization methods, we found that all methods generally provided similar geometries, with the exception of GFN-FF/ALPB, which gave somewhat inferior poses as seen on worse RMSD.

The docking results were also encouraging from the standpoint of universality across the ligand chemotypes. For disaccharide-based ligands, we found that both sugar units are reliably being docked into their native binding positions. Better still, the aryltriazolyl decorations, as well as other aromatic decorations that are a frequent motive in galectin inhibitors, are also consistently being docked into the Arg144-defined pocked that is expected by the 5H9P structure. We were also able to analyze docked poses of two ligands that interact with Gal-3 via a halogen bond (GFA_17 and GFA_52), for which crystal structures have been published. We found that for both of these ligands, the halogen atom is docked very close to the crystal position, and that this position is maintained during pose optimizations. Although these results appear promising, a more systematic investigation that is outside the scope of this manuscript is needed to assess the ability of docking algorithms to predict geometries of halogen bonds in galectin inhibitors.

### Overall Evaluation of the Tested Methods

To evaluate the ability of the tested methods to rank ligands in the GFA ligand set, we use two quantities that aim to estimate protein–ligand affinity. First, the binding enthalpy Δ*H*_bind_ captures the enthalpic contributions, namely the interaction energy, the desolvation energy and the ligand conformational strain. Second, the binding free energy Δ*G*_bind_ also includes entropic contribution calculated from harmonic frequency analysis in the modified rigid-rotor-harmonic-oscillator (RRHO) approximation. Importantly, all terms of the Δ*H*_bind_ score are calculated by each tested method separately and Δ*H*_bind_ thus provides a direct comparison between the methods in all enthalpic terms. On the other hand, the entropic term used for Δ*G*_bind_ was calculated by the GFN2 method, which is perhaps the highest level of theory available in the context of this study. In other words, the Δ*G*_bind_ score is constructed from Δ*H*_bind_ calculated at the respective level for each method and the entropic term  calculated by GFN2. As such, the Δ*G*_bind_ score is physically more complete as it includes the loss of translational, rotational and vibrational entropy, which is an important part of binding.

**Figure 4:**
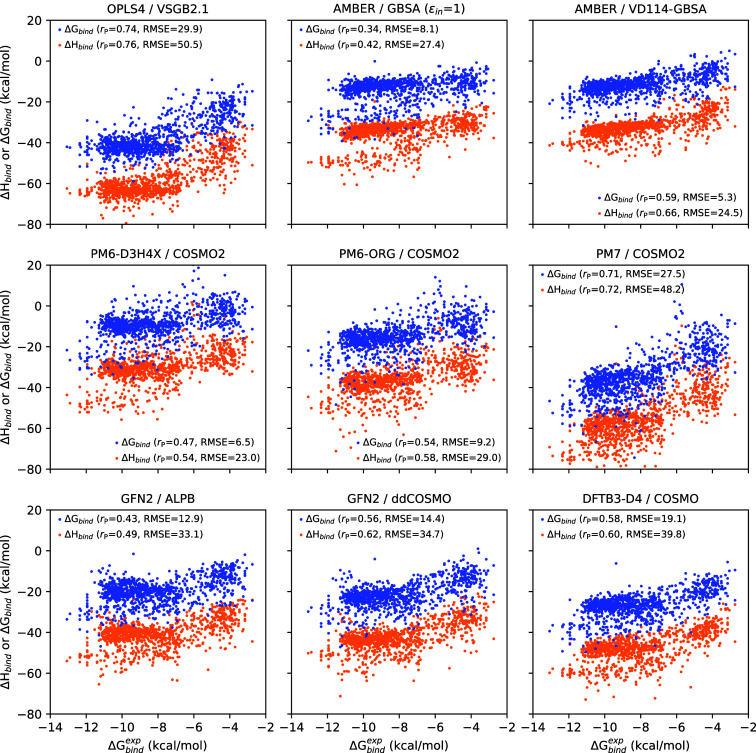
Correlation between the computed quantities Δ*H*_bind_ or Δ*G*_bind_, and experimental Δ*G*_bind_^exp^ for the most successful methods.

Table 2 shows the ability of the Δ*H*_bind_ and Δ*G*_bind_ scores, as calculated by the different methods, to reproduce the experimental affinities Δ*G*_bind_^exp^ in terms of Pearson’s and Spearman’s correlation coefficients (*r*_P_ and ρ) and RMSE. Collectively, our results show that while the Δ*G*_bind_ score leads to a worse correlation with experiment compared to Δ*H*_bind_, it significantly improves RMSE. The improvement in RMSE is attributed to the entropic penalty to binding caused by the loss of degrees of freedom, which is captured in Δ*G*_bind_ but not in Δ*H*_bind_. This entropic penalty accounts for roughly 20–25 kcal/mol and thus the Δ*H*_bind_ and Δ*G*_bind_ scores are shifted by this value (Figure 4). Unfortunately, the inclusion of entropy in Δ*G*_bind_ also led to increased noise and hence a decrease in correlation metrics, which was quite significant for most methods. All in all, one can argue that the Δ*H*_bind_ score performs better if correlation with experiment is a priority, but the Δ*G*_bind_ score is clearly more meaningful if absolute values of binding free energy are the goal.

**Table 2:** Correlation and Regression Metrics between Computed Quantities Δ*H*_bind_ or Δ*G*_bind_, and Experimental Δ*G*_bind_^exp^

		Δ*H*_bind_			Δ*G*_bind_		
method type	method	*r*_P_	ρ	RMSE (kcal/mol)	*r*_P_	ρ	RMSE(kcal/mol)
scoring function	glide SP	-	-	-	0.29^a^	0.23^a^	3.54^a^
force field	OPLS4/VSGB2.1	0.76	0.63	50.53	0.74	0.62	29.98
	OPLS 2005/VSGB2.1	0.77	0.64	67.56	0.76	0.63	46.91
	AMBER/GBSA (ε_in_ = 1)	0.42	0.46	27.36	0.34	0.35	8.05
	AMBER/GBSA (ε_in_ = 4)	0.58	0.54	33.16	0.54	0.52	12.94
	AMBER/VD114-GBSA	0.66	0.65	24.48	0.59	0.58	5.25
	AMBER/VAD-GBSA	0.55	0.50	31.36	0.51	0.48	10.94
	GFN-FF/ALPB	0.40	0.41	41.15	0.34	0.37	20.80
PMx	PM6-D3H4/COSMO	0.31	0.22	21.82	0.24	0.15	7.65
	PM6-D3H4X/COSMO	0.29	0.20	21.65	0.22	0.13	7.74
	PM6-ORG/COSMO	0.36	0.31	23.22	0.31	0.24	6.92
	PM7/COSMO	0.63	0.61	46.16	0.62	0.60	25.52
	PM6-D3H4/COSMO2	0.55	0.53	23.19	0.49	0.45	6.50
	PM6-D3H4X/COSMO2	0.54	0.51	23.01	0.47	0.43	6.49
	PM6-ORG/COSMO2	0.58	0.57	28.95	0.54	0.53	9.22
	PM7/COSMO2	0.72	0.67	48.18	0.71	0.68	27.47
xTB	GFN1/GBSA	0.50	0.45	33.20	0.45	0.38	13.00
	GFN1/ALPB	0.50	0.44	38.31	0.48	0.41	17.88
	GFN2/GBSA	0.56	0.49	32.25	0.48	0.40	12.08
	GFN2/ALPB	0.49	0.39	33.09	0.43	0.33	12.90
	GFN2/ddCOSMO	0.62	0.53	34.68	0.56	0.48	14.39
DFTB	DFTB3-D3H5/COSMO	0.54	0.51	53.99	0.54	0.50	33.08
	DFTB3-D4/COSMO	0.60	0.54	39.76	0.58	0.52	19.14

a Based on the Glide SP scoring function instead of Δ*G*_bind_.

Focusing on the comparison between MM and SQM levels of theory, it is apparent that the SQM methods did not systematically overperform the force fields in neither correlation nor regression metrics in this analysis. In fact, the best methods from both MM and SQM levels achieved comparable correlation with experiment (*r*_P_ around 0.6–0.8), which we consider to be the most important measure of success. When measured by *r*_P_, the best overall results were achieved by the OPLS4 and OPLS 2005 force fields coupled with the VSGB2.1 solvation model, closely followed by PM7/COSMO2, AMBER/VD114-GBSA, GFN2/ddCOSMO, and DFTB3-D4/COSMO.

From MM methods, both the OPLS4 and OPLS 2005 force fields showed very similar performance and were both better than the other force fields that we tested. These results seem to agree with a recent investigation, where OPLS was found to be the best-performing force field in the context of alchemical free energy calculations.^77^ Interestingly, the AMBER/GBSA method performed quite poorly with the solute interior dielectric constant set to the default value of ε_in_ = 1, but improved significantly with higher values of ε_in_. Specifically, using ε_in_ = 4 led to improved *r*_P_ from 0.42 to 0.58 for the Δ*H*_bind_ score. We also tested two solvation regimes where ε_in_ is not uniform but varies in a residue- or atom-specific manner. We found that the VD114-GBSA^58^ model, where a value of ε_in_ = 4 is assigned only to charged protein atoms, exhibited even better performance (*r*_P_ = 0.66). Alternatively, the VAD-GBSA^59^ model, where ε_in_ is assigned specifically to each atom of both protein and ligand, showed slightly worse performance (*r*_P_ = 0.55) than VD114-GBSA, but was still better than the default setting of ε_in_ = 1. It is interesting to note that both the VD114-GBSA^58^ and VSGB2.1^56^ models, which are both among the most successful solvation models in our investigation, use ε_in_ values higher than 1 (up to 4) for highly polarizable protein atoms. Our results confirm that this modification is advantageous in GB-based solvation models, even though its theoretical basis is rather heuristic. We also tested the GFN-FF/ALPB method implemented in xTB, which achieved a similar performance as AMBER/GBSA with ε_in_ = 1.

Looking at the SQM methods, it is apparent that the results vary significantly depending on the solvation model used. This fact shows that the results should not be viewed only in terms of the SQM Hamiltonian itself, but should be judged in combination with a given method for solvation energy calculation. The influence of the solvation model is prominent for the PMx methods coupled with COSMO-based solvation. It is clear that for all the tested PM6 and PM7 variants, the updated COSMO2 model significantly outperformed the default COSMO implementation in MOPAC. While this increase in performance was most pronounced for the PM6-based methods (*r*_P_ = 0.36 vs 0.58 for PM6-ORG coupled with COSMO vs COSMO2, respectively), it was apparent in the case of PM7 as well (*r*_P_ = 0.63 vs 0.72 coupled with COSMO vs COSMO2, respectively) when quantified for Δ*H*_bind_. In fact, PM7 combined with both COSMO and COSMO2 achieved a higher overall *r*_P_ than all the other tested SQM methods. The tested variants of PM6, namely PM6-D3H4, PM6-D3H4X, and PM6-ORG, all performed comparably, but PM6-ORG had a slight advantage (*r*_P_ = 0.55, 0.54, and 0.58 respectively, when coupled with COSMO2).

The tight-binding methods performed relatively similarly, although we are unable to draw an apples-to-apples comparison between the DFTB and xTB formalisms, as consistent calculations with the same solvation model were not available. From xTB methods, both GFN1 and GFN2 provided similar results (*r*_P_ around 0.50). The legacy GBSA and the newer ALPB solvation models also lead to quite similar results (*r*_P_ = 0.50 vs 0.50 with GFN1 and 0.56 vs 0.49 with GFN2). However, a markedly better result (*r*_P_ = 0.68) was obtained when GFN2 was coupled with ddCOSMO solvation. The difference in performance between the simpler GB or ALPB models and the more elaborate ddCOSMO scheme for the GFN2 method again highlights the importance of the solvation model. From DFTB methods, both DFTB3-D3H5 and DFTB3-D4 coupled with COSMO (DFTB+ implementation) scored similarly, with a slight advantage of DFTB3-D4 (*r*_P_ = 0.54 vs 0.60, respectively).

### Subset-Wise Decomposition

**Figure 5:**
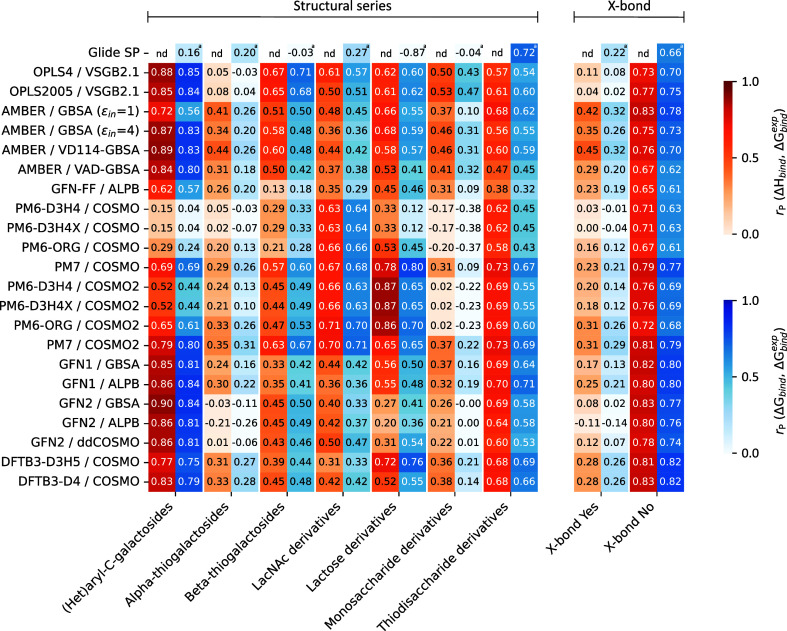
Performance of the tested methods for different subsets of the GFA ligand set. Orange scale indicates correlation between calculated Δ*H*_bind_ and Δ*G*_bind_^exp^. Blue scale indicates correlation between calculated Δ*G*_bind_ and Δ*G*_bind_^exp^. Subsets are defined according to the individual ligand series (left) or the presence of putative halogen bonding (right). ^a^Based on the Glide SP scoring function instead of Δ*G*_bind_; nd = not determined.

While the overall correlation across all ligands is revealing, it is also interesting to look at the subset-wise performance of the tested methods. We first investigated the correlation with experimental affinities within the ligand series defined in the GFA ligand set (Figure 5, left panel). The results show that some series were consistently scored poorly whereas ligands in some other series were easier to rank. Prominently, the series of *Alpha-thiogalactosides* was scored particularly poorly by most methods. This result is not surprising, as this series includes most of the ligands with putative halogen bonding, which generally led to worse results in this study as discussed later. However, there were also differences between methods in their ability to score certain ligand series. For instance, the series of *Lactose derivatives* was scored very well by all the PM6-based methods coupled with the COSMO2 model, but it was scored poorly by GFN2 coupled with GBSA and ALPB solvation models. Conversely, the series of *(Het)aryl-C-galactosides* was scored very well by most methods, but the performance of the PM6-based methods in this series was worse. Even more interestingly, considering that the COSMO solvation model generally achieved much worse results than COSMO2 in our study, it is peculiar that for both series of *LacNAc derivatives* and *Thiodisaccharide derivatives*, COSMO performed very similarly to COSMO2, whereas the results for all other series worsened significantly.

Interesting insights are obtained by isolating the contribution of ligands with putative halogen bonding (Figure 5, right panel). We found that if halogen-bonding ligands are removed from the set, all methods achieve dramatically better *r*_P_ with very little differences between them. Thus, the inclusion of this subset of ligands is a major source of error for all the tested methods. It is quite disappointing that the halogen-bonding subset was not scored systematically better by SQM methods, which should in principle be better at handling anisotropic phenomena such as σ-hole interactions, than by force fields. In fact, the best *r*_P_ on this subset was achieved by the AMBER-based methods. In addition, we found that SQM methods containing additional empirical corrections for halogen bonding, namely PM6-D3H4X and GFN1, did not provide improved performance on this subset compared to other SQM methods. Although we note that during the preparation of this paper, a new parametrization of the X correction for PM6 was published,^9^ but has not yet been implemented in MOPAC. The reasons for the inconsistent scoring of the halogen-bonding subset of ligands are not fully clear from our data, although we expect this ligand set to be well-suited for further investigation of this problem.

**Figure 6:**
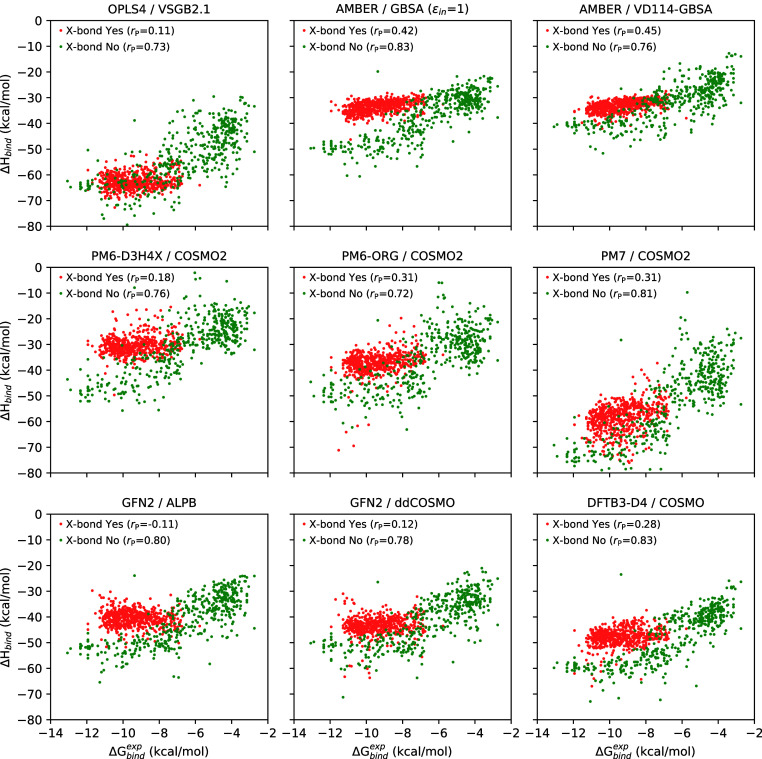
Inconsistent scoring of ligand subsets with and without putative halogen bonding shown by correlation between the computed Δ*H*_bind_ and experimental Δ*G*_bind_^exp^.

The decomposition of the results according to the presence of a putative halogen bond for the best-performing methods is further shown in Figure 6 as scatter plots between Δ*H*_bind_ and Δ*G*_bind_^exp^. It is quickly apparent that scoring the subsets with (red) and without (green) halogen bonding consistently against each other is a major challenge. It is also important to note that this inconsistency has a significant impact on the overall correlation metrics between model and experiment when quantified across the entire ligand set. These results highlight the importance of testing computational methods on diverse and noncongeneric series of ligands to determine the true accuracy of the predictions. Overall, these detailed decomposed results show that the scoring performance of the methods tested here is not monolithic across the chemical space of the ligand set, but rather depends on trackable differences in the ligand chemotypes. These differences also likely hold clues for the optimization of future computational models.

### Decomposition into Individual Energy Terms

**Figure 7:**
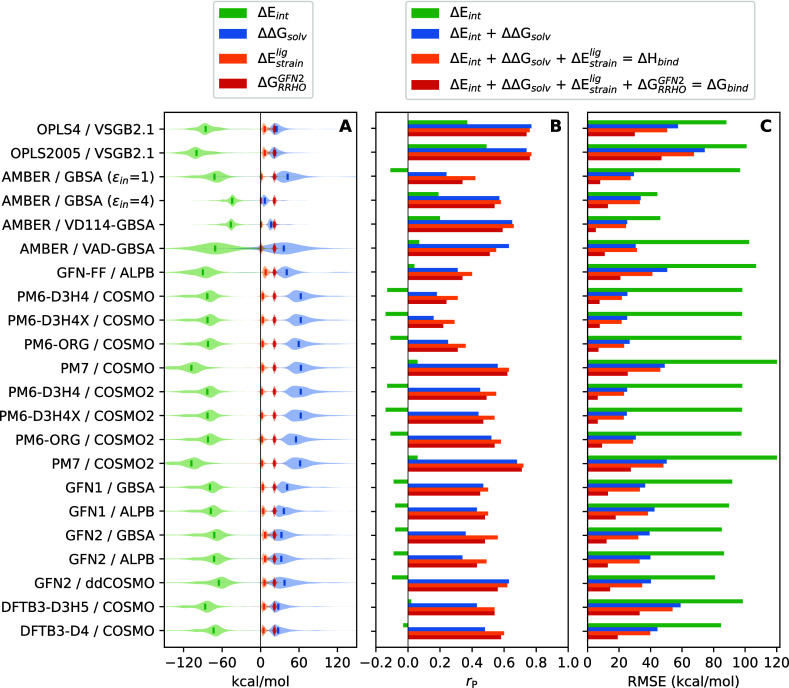
Decomposition into individual affinity terms. (A) Δ*E*_int_, ΔΔ*G*_solv_, , and  terms as computed by different methods. Analysis includes all 10,849 poses obtained from docking. Solid lines inside the violins indicate median values. (B) Contributions of the individual terms to the overall correlation with experiment. (C) Contributions of individual terms to RMSE with experiment. For more details see Figures S4–S7 in the Supporting Information.

To decipher the observed differences in the performance of the tested methods, we decomposed the computed affinities into individual terms and analyzed them. The results of this analysis are summarized in Figure 7. At first glance, it is apparent that the individual energy terms computed by different methods differ in magnitude (Figure 7A). As stated earlier, the main enthalpic terms evaluated by our scoring scheme are Δ*E*_int_ and ΔΔ*G*_solv_. These two terms have similar absolute value but opposite sign, which means that they largely compensate each other. The enthalpic compensation between Δ*E*_int_ and ΔΔ*G*_solv_ could be thought of as a competition between the ligand’s affinity toward the binding pocket or toward water. Both of these enthalpic terms are dominated by electrostatics and depend on atomic partial charges computed by a given method. These charges are used to calculate the electrostatic contribution to the interaction energy and are also passed to the implicit solvation model for the calculation of the electrostatic part of the solvation energy. The  term is much smaller in magnitude (usually less than 10 kcal/mol) and provides a relatively small unfavorable correction to binding. Finally, the entropic term  provides a relatively large unfavorable penalty (approximately 20–25 kcal/mol) which further compensate the favorable contribution of Δ*E*_int_.

Several observations can be drawn from our decomposition analysis. Regarding the AMBER-based methods, using ε_in_ = 4 or the VD114 model resulted in significantly less negative Δ*E*_int_ and less positive ΔΔ*G*_solv_. This is caused by the higher ε_in_ being passed into the GB solvation calculation, as well as in the force field Coulomb term, which is used for calculating Δ*E*_int_. It is interesting to note that despite this alteration, these methods performed better than when using the default ε_in_ = 1 setting. In fact, the AMBER/VD114-GBSA method was among the best-performing methods overall. The VAD-GBSA model clearly leads to a wider spread in the calculated Δ*E*_int_ and ΔΔ*G*_solv_ values, which may contribute to its inferior performance compared to VD114-GBSA. Focusing on the SQM methods, the most striking observation is that PM7 provides markedly more negative Δ*E*_int_ values compared to other SQM methods. This observation is in agreement with a previous benchmarking study,^5^ which also found that PM7 provided more negative values of interaction energy on a PLA15 benchmark, when compared to QM level. Interestingly, the values of ΔΔ*G*_solv_ computed by COSMO models based on PM7 are of comparable magnitude to those computed with PM6. This disparity then results in more favorable Δ*H*_bind_ computed by PM7. On another note, we have also observed that the ΔΔ*G*_solv_ term computed by MOPAC implementation of COSMO is significantly shifted toward positive values, and thus provides more unfavorable contribution to Δ*H*_bind_. This is unlikely to be an effect of the COSMO formalism itself, as it was not observed for ΔΔ*G*_solv_ energies calculated by DFTB+ implementation of COSMO, and neither for ddCOSMO implementation in xTB. This effect is more likely caused by parametrization of the model (e.g., atomic radii), which differs in different implementations.

Other interesting remarks can be made when decomposing the contributions of the individual terms to the overall correlation with experimental affinities (Figure 7B). For OPLS-based methods, we found that a large part of correlation was already achieved by considering the Δ*E*_int_ term alone (7B, green bars). In contrast, the Δ*E*_int_ term alone has almost no correlation when computed by SQM methods. This indicates that in the OPLS-based models, the affinity is already to a large extent encoded in the force field-derived Δ*E*_int_ term, whereas the SQM-based methods achieve the correlation more realistically by a compensation between Δ*E*_int_ and ΔΔ*G*_solv_. Focusing on , we found that the inclusion of this term (7B, orange bars) improved correlation with experimental affinities in most cases, but the improvement was more consistent with the SQM methods. These results seem to indicate that the SQM level of theory is superior to force fields in describing of conformational strain. In fact,  was of very little benefit or even worsened the results with some of the force field that we tested. Finally, the inclusion of the entropic  term led to slightly deteriorated correlation with experimental affinities.

Focusing on the effect of the individual terms on RMSE, it is clear that the inclusion of each term successively improves RMSE (Figure 7C). These findings highlight the physicality of this scoring approach, where each term brings the final score closer to the experimental values. It is especially noteworthy to point out the contribution of the  term (7C, red bars), which helps significantly to bring the final score closer to the realistic values of the actual binding free energy. Yet entropic terms are often missing in MM/(PB)GBSA or similar workflows. Clearly, the development of efficient and accurate approaches to calculate entropy loss in protein–ligand binding should be of high priority for absolute binding free energy predictions.

### Decomposition of Solvation Energy

We further decomposed the calculated ΔΔ*G*_solv_ term into different solvation contributions. The most important solvation term is the polar (electrostatic) contribution, which corresponds to the energy of embedding the molecular system in a dielectric continuum. In the VSGB2.1 solvation model in Schrödinger Prime, polar solvation is modeled by a surface GB term^78^ with variable ε_in_ for polar protein residues.^56^ In the AMBER methods, we used either the default GB model with ε_in_ set to 1 or 4, or alternatively the variable-dielectric models VD114-GBSA^58^ and VAD-GBSA.^59^ From the SQM methods used in this study, the polar contribution is modeled either by COSMO^23,72^ implementations in MOPAC and DFTB+, the ddCOSMO^64^ implementation in xTB, or by GB^21,79^ or analytical linearized Poisson–Boltzmann (ALPB)^62,80^ terms computed in xTB.

Some models also attempt to include the nonpolar solvation energy, which should, in principle, encompass the cavitation energy, as well as the solvent–solute VdW attraction. Specifically, both GBSA in AMBER and the COSMO2 models use a molecular SASA term, where the molecular surface area is scaled globally by a surface tension parameter.^41^ Similarly, SASA term is also modeled in xTB, but the surface tension parameters are element-specific.^62^ The VSGB2.1 model attempts to model the nonpolar solvation indirectly by including an empirical hydrophobic term, which rewards close contacts of nonpolar heavy atoms.^56^ Additionally, the GBSA and ALPB solvation models in xTB also include a rudimentary description of solute–solvent hydrogen bonding by a SASA-dependent approximate Keesom interaction term, where the hydrogen bond strength is tabulated in element-specific manner, and dipole moments are approximated by squares of atomic partial charges.^62^ For the purpose of this analysis, we also added this term to the ddCOSMO model. Table 3 provides an overview of the implicit solvation models used in this study.

**Table 3:** Overview of Implicit Solvation Models Employed in This Study

model	program	polar term	nonpolar term	HB term	Ref
VSGB2.1	Schrödinger Prime	S-GB with variable ε_in_	empirical hydrophobic	-	(56,78)
GBSA (ε_in_ = 1)	AMBER	GB with ε_in_ = 1	molecular SASA	-	-
GBSA (ε_in_ = 4)	AMBER	GB with ε_in_ = 4	molecular SASA	-	-
VD114-GBSA	AMBER^a^	GB with variable ε_in_	molecular SASA	-	(58)
VAD-GBSA	AMBER^a^	GB with atom-wise variable ε_in_	molecular SASA	-	(59)
COSMO	MOPAC	COSMO	-	-	(72)
COSMO	DFTB	COSMO	-	-	(72)
COSMO2	MOPAC^b^	COSMO′ (Řezáč radii)	molecular SASA	-	(41)
GBSA	xTB	GB	atom-wise SASA	approx. Keesom	(18)
ALPB	xTB	ALPB	atom-wise SASA	approx. Keesom	(62)
ddCOSMO	xTB	ddCOSMO	atom-wise SASA	approx. Keesom^c^	(64)

a VD114 and VAD models with variable dielectric regimes were calculated using a customized MMPBSA.py script in AMBER18.

b MOPAC was used for calculating the polar solvation energy using modified atomic radii. The molecular SASA term was calculated outside of MOPAC.

c HB term is not by default included in the ddCOSMO implementation in xTB. It was added to the ddCOSMO model as calculated by GFN2/ALPB.

**Figure 8:**
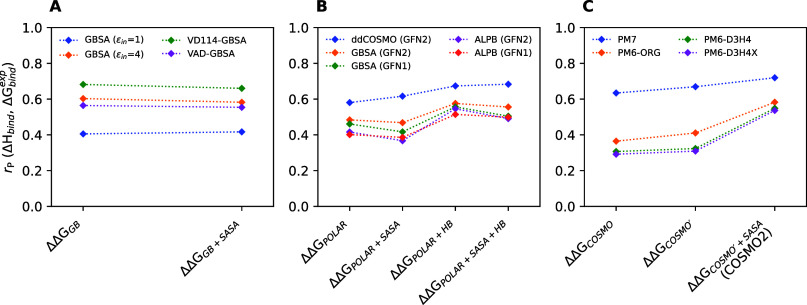
Effect of individual solvation terms on correlation between Δ*H*_bind_ and experimental affinity Δ*G*_bind_^exp^ for (A) AMBER-based models, (B) MOPAC-based models, and (C) xTB-based models. ΔΔ*G*_COSMO’_ refers to the COSMO energy from MOPAC calculated with the Řezáč radii.^41^

**Figure 9:**
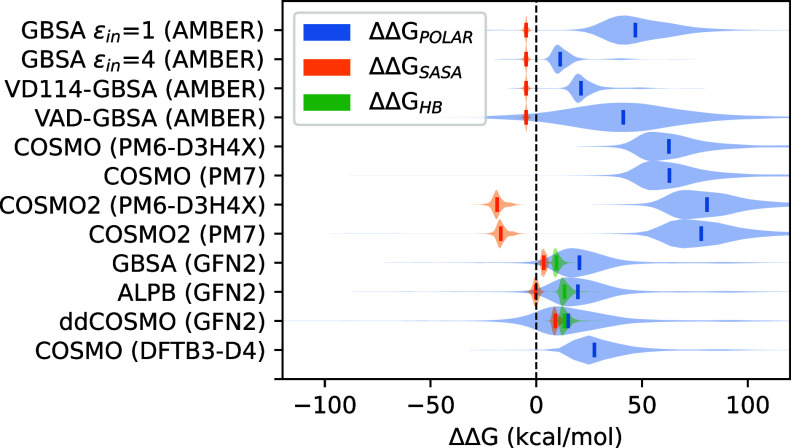
Decomposition of solvation energies into individual solvation terms. ΔΔ*G*_POLAR_ refers to the polar part of the solvation energy calculated by the given model. Analysis includes all 10,849 poses obtained from docking. Solid lines inside the violins indicate median values. For more details, see Figure S8 in the Supporting Information.

The decomposition of solvation contributions provided several important observations. First, as shown in Figure 8A, the inclusion of ΔΔ*G*_SASA_ in the AMBER-based methods did not lead to any improvement in accuracy. Similarly, as depicted in Figure 8B, using the atom-wise ΔΔ*G*_SASA_ in xTB-based models did not provide substantial improvement in correlation either. In fact, it worsened the correlation for the GBSA and ALPB models, whereas it only marginally improved correlation for ddCOSMO. On the other hand, contrasting results were obtained when examining the differences between the original COSMO parametrization in MOPAC and the updated COSMO2 model, which performed much better in this study (Figure 8C). The improved correlation achieved by COSMO2 does not result from the polar energy calculated using the newly reparametrized atomic radii (which we call ΔΔ*G*_COSMO’_), but actually comes from the newly added molecular SASA term. On closer inspection, we found that ΔΔ*G*_SASA_ calculated by COSMO2 yields much more negative values (approximately −20 kcal/mol) compared to ΔΔ*G*_SASA_ calculated by AMBER (approximately −5 kcal/mol) or by xTB (approximately 0–10 kcal/mol), as shown in Figure 9. Thus, the improvement achieved by adding the SASA term to COSMO2 stems from fairly large favorable correction to the calculated solvation energy. While this correction is obviously achieved by a SASA-based term, it is unclear if it could actually be physically tied to nonpolar solvation. Interestingly, the solvent–solute hydrogen bonding term ΔΔ*G*_HB_ implemented in xTB was found to provide substantial benefits for all xTB solvation models (Figure 8B). Looking at this contribution in more detail, ΔΔ*G*_HB_ contributes unfavorably to binding in our set with a magnitude of approximately 10 to 15 kcal/mol (Figure 9). Collectively, our results support the use of ΔΔ*G*_HB_ in implicit solvent models in the context of protein–ligand affinity prediction.

## Conclusions

In this study, we gathered a large set of galectin inhibitors from literature and meticulously categorized it into several subsets and structural series. Using this set, we have conducted a comprehensive evaluation of several classical and semiempirical quantum mechanical (SQM) potentials to compare their ability to rank the inhibitors according to their affinity. We included the most state-of-the-art potentials up to the tight-binding level combined with available implicit solvation models. As the main conclusion, we found that using the more expensive SQM potentials did not alone lead to consistently better results compared to the force field methods. This result should not discourage the use of SQM potentials for protein–ligand scoring, but rather highlights the limited value of increasing the accuracy of interaction energy calculation alone without addressing other energetic terms that govern protein–ligand binding. The free energy change associated with binding is a result of intricate compensation between several physical phenomena which all need to be estimated accurately to achieve better prediction. We observed major variations in ranking accuracy when using different solvation models at both MM and SQM levels. For some methods, the use of different solvation models accounted for up to a 50% difference in accuracy, as measured by correlation with experimental affinities. There were also important differences in the ability of the tested methods to rank ligands correctly within individual subsets, but also to compare the subsets against each other on a consistent affinity scale. This was particularly challenging when comparing subsets of ligands with and without putative halogen bonding. The inclusion of an entropic RRHO term corresponding to the loss of translational, rotational, and vibrational degrees of freedom introduces an important physical penalty to binding, which brings the calculated affinity closer to the experimental affinities. However, this term, even when calculated relatively expensively at the level of extended tight-binding (GFN2), also brings quite a noticeable noise into the final score which deteriorates the correlation with experiment. Our investigation provides a comparison of the most modern end-point methods for scoring protein–ligand affinities on a large real-world set of ligands. The GFA ligand set is released together with this paper and we expect it to become a convenient proving ground for the development of future computational models.

## Data Availability

The GFA ligand set together with docked and optimized geometries is available on GitHub (https://github.com/choutkaj/GFA-ligand-set). An archived version is also available on Zenodo via https://doi.org/10.5281/zenodo.14585059.

## Supporting Information Available

Additional figures (PDF)
GFA ligand set including the ligands in SMILES strings and all the necessary information about the ligands (XLSX)
Energies for all poses and for best poses per ligand computed by all evaluated methods (XLSX)
