# Supplementary material for: End-Point Affinity Estimation of Galectin Ligands by Classical and Semiempirical Quantum Mechanical Potentials
Source: J Chem Inf Model. 2025 Jan 4;65(2):762–77. doi: 10.1021/acs.jcim.4c01659 (PMC11776057; doi:10.1021/acs.jcim.4c01659)
Supplement: Supplementary file 1 — ci4c01659_si_001.pdf [file ci4c01659_si_001.pdf]

SUPPORTING INFORMATION

End-Point Affinity Estimation of Galectin Ligands by Classical and Semiempirical Quantum Mechanical Potentials

Jan Choutka,*[1] Jakub Kaminský,[1] Ercheng Wang,[2] Kamil Parkan,[1,3] Radek Pohl[1]

[1] Institute of Organic Chemistry and Biochemistry of the Czech Academy of Sciences, Gilead Sciences & IOCB Research Centre, Flemingovo nám. 2, 166 10, Prague, Czech Republic

[2] Zhejiang Laboratory, Hangzhou, 311100, China

[3] Department of Chemistry of Natural Compounds, University of Chemistry and Technology Prague, Technická 5, 166 28, Prague, Czech Republic

E-mail: jan.choutka@uochb.cas.cz

Additional Figures

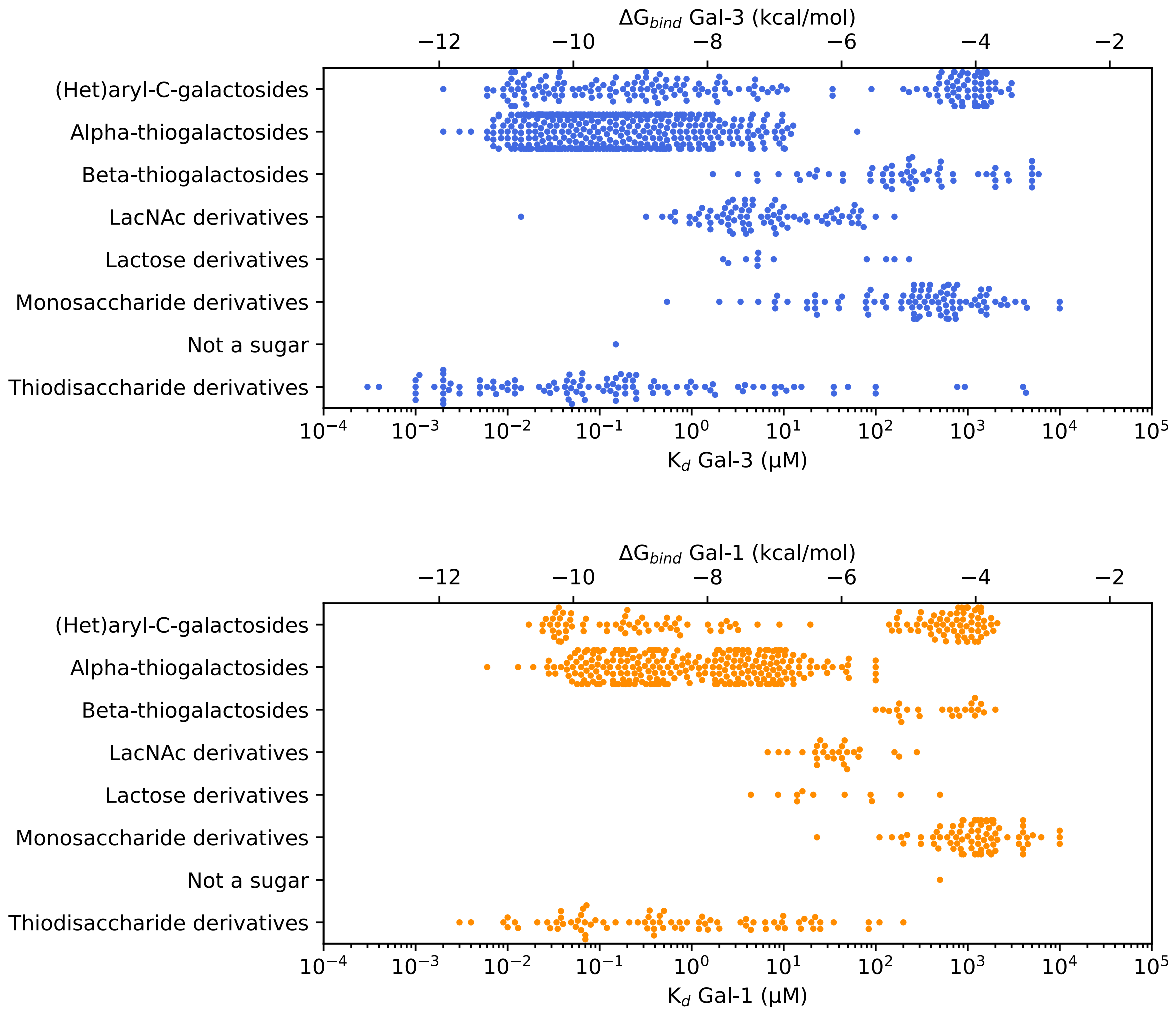

Figure S1. Distribution of affinities in the GFA ligand set for Gal-3 (top) and Gal-1 (bottom).

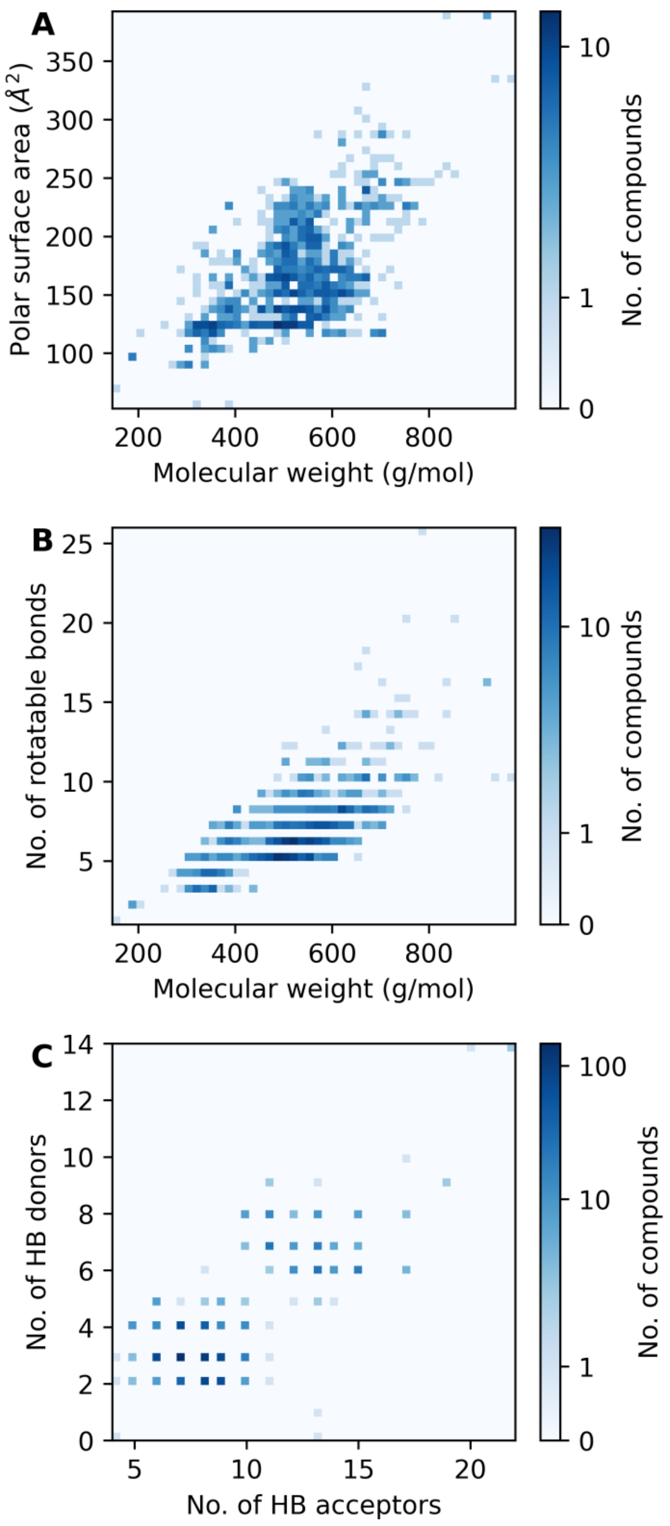

Figure S2. Distribution of physicochemical properties in the GFA ligand set. (A) Distribution of molecular weight and polar surface area. (B) Molecular weight and number of rotatable bonds. (C) Numbers of hydrogen bond acceptors and donors.

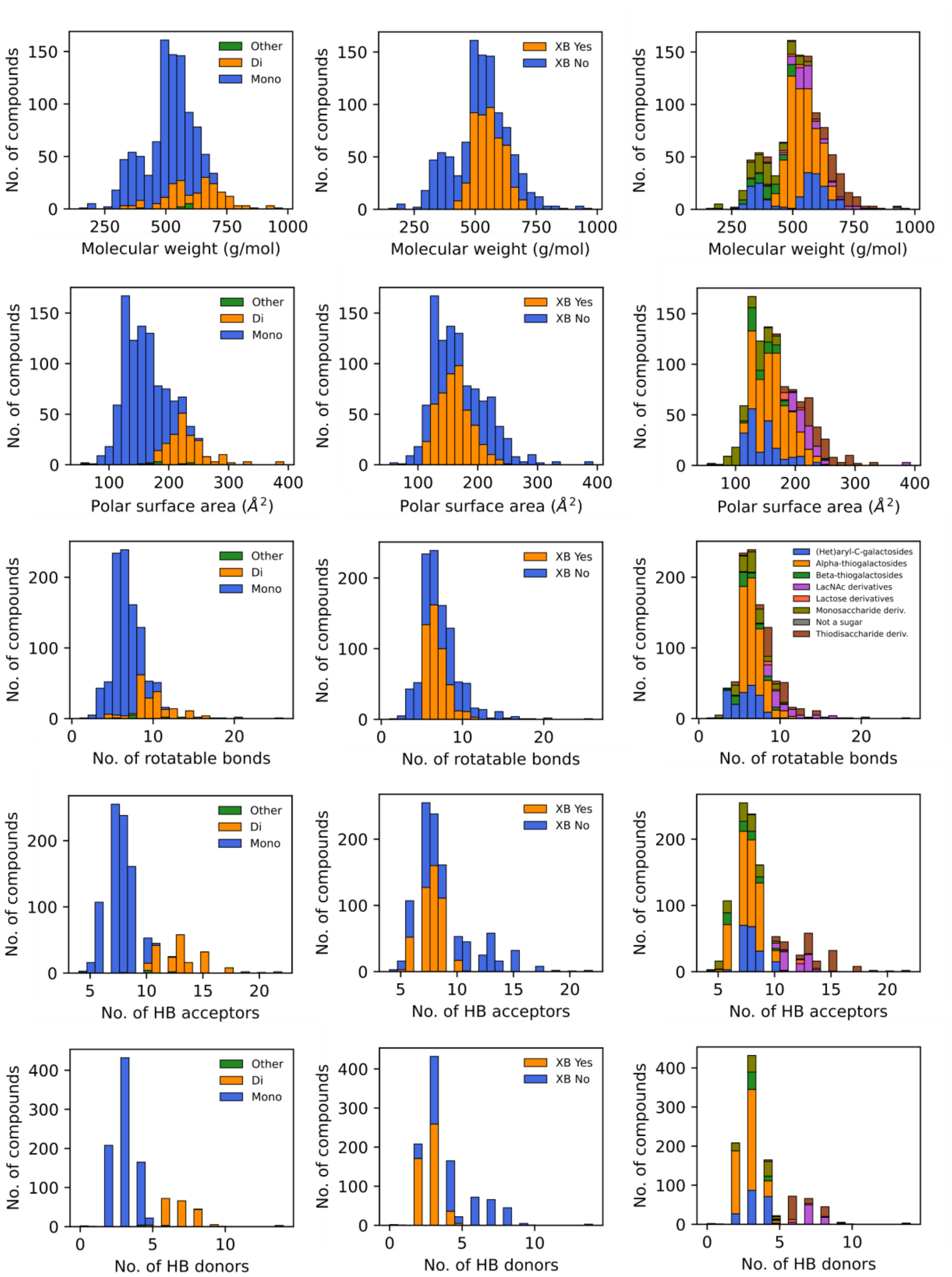

Figure S3. Subset-wise histograms of physicochemical properties in the GFA ligand set.

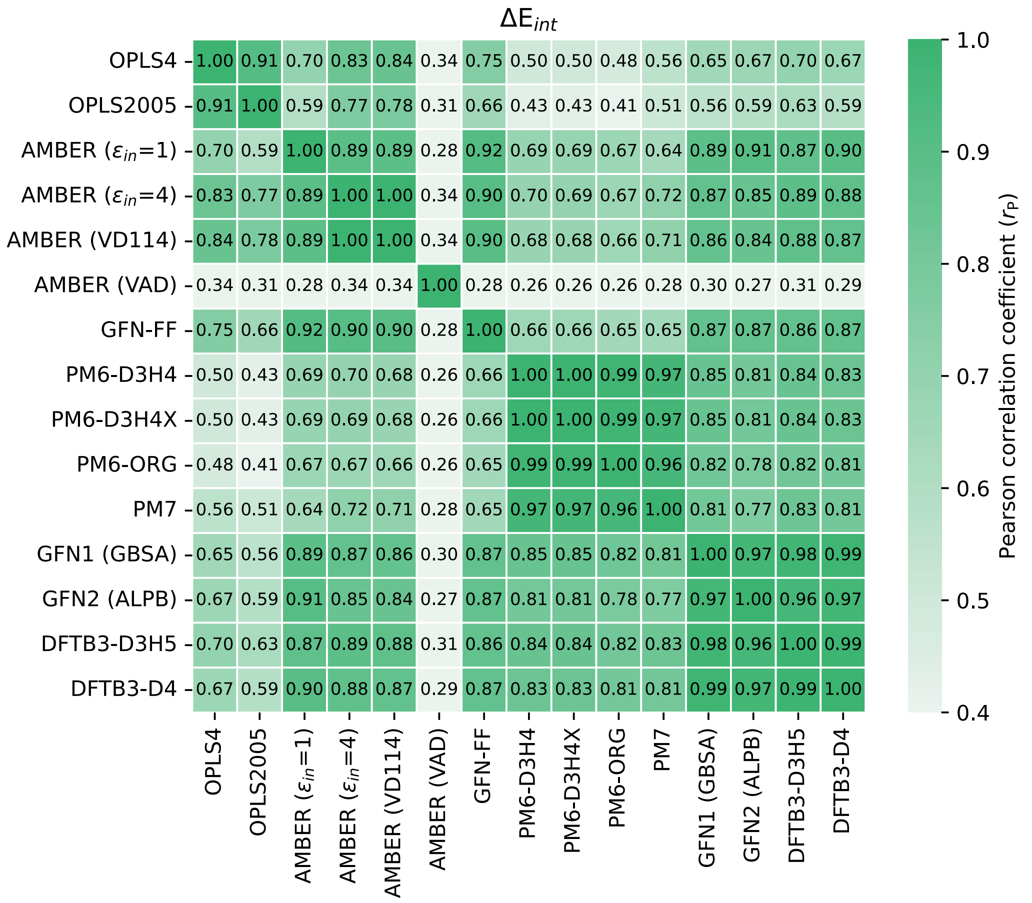

Figure S4. Heatmap of Pearson correlation coefficients for the ΔE_{int} term as computed by different methods. All 10849 poses are included in the analysis.

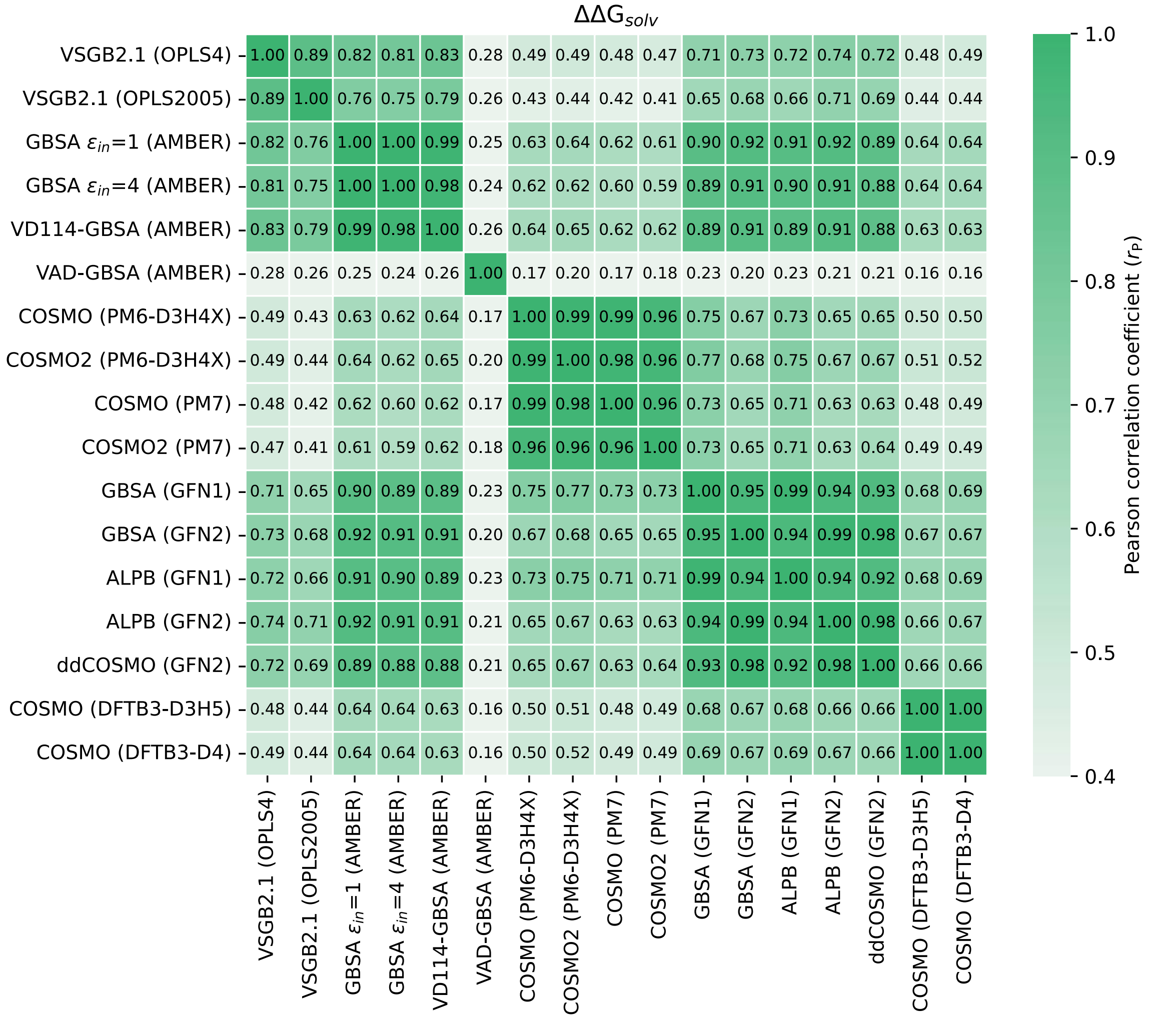

Figure S5. The same as S3, but for $\Delta\Delta G_{solv}$ term.

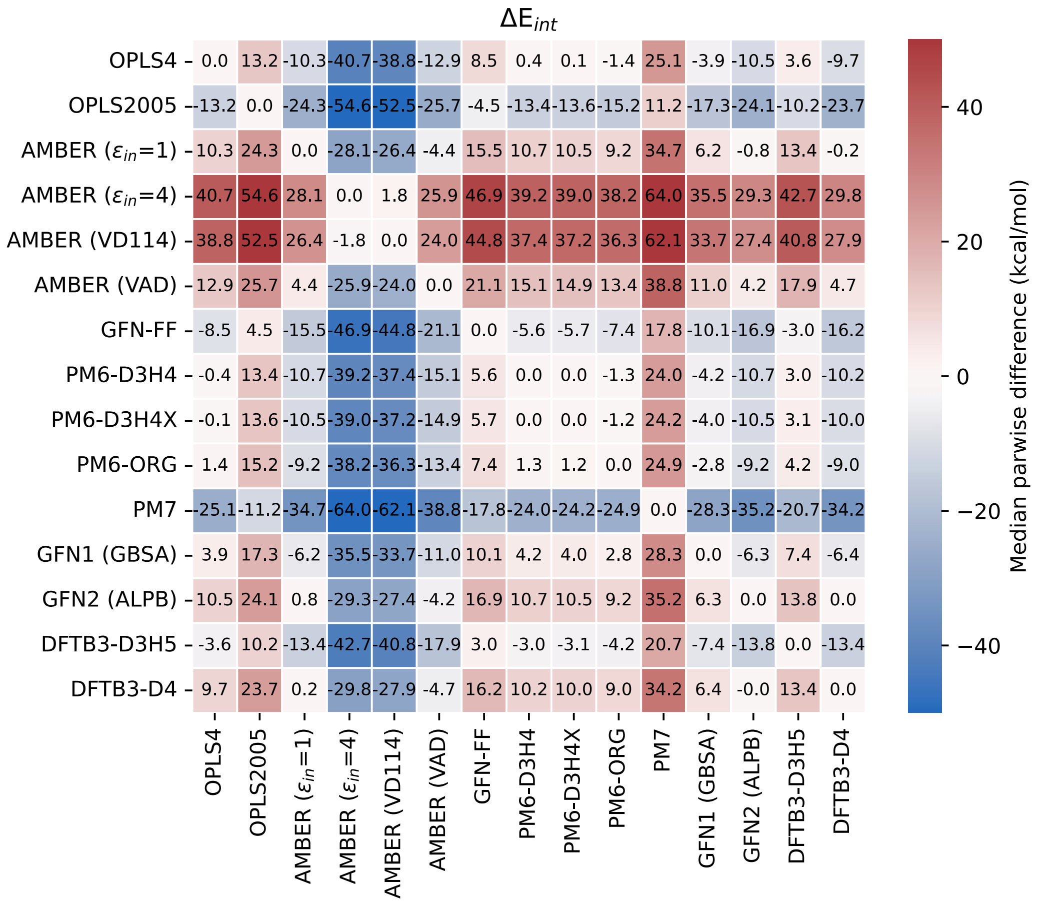

Figure S6. Heatmap of median pairwise differences for the ΔE_{int} term as computed by different methods. All 10849 poses are included in the analysis.

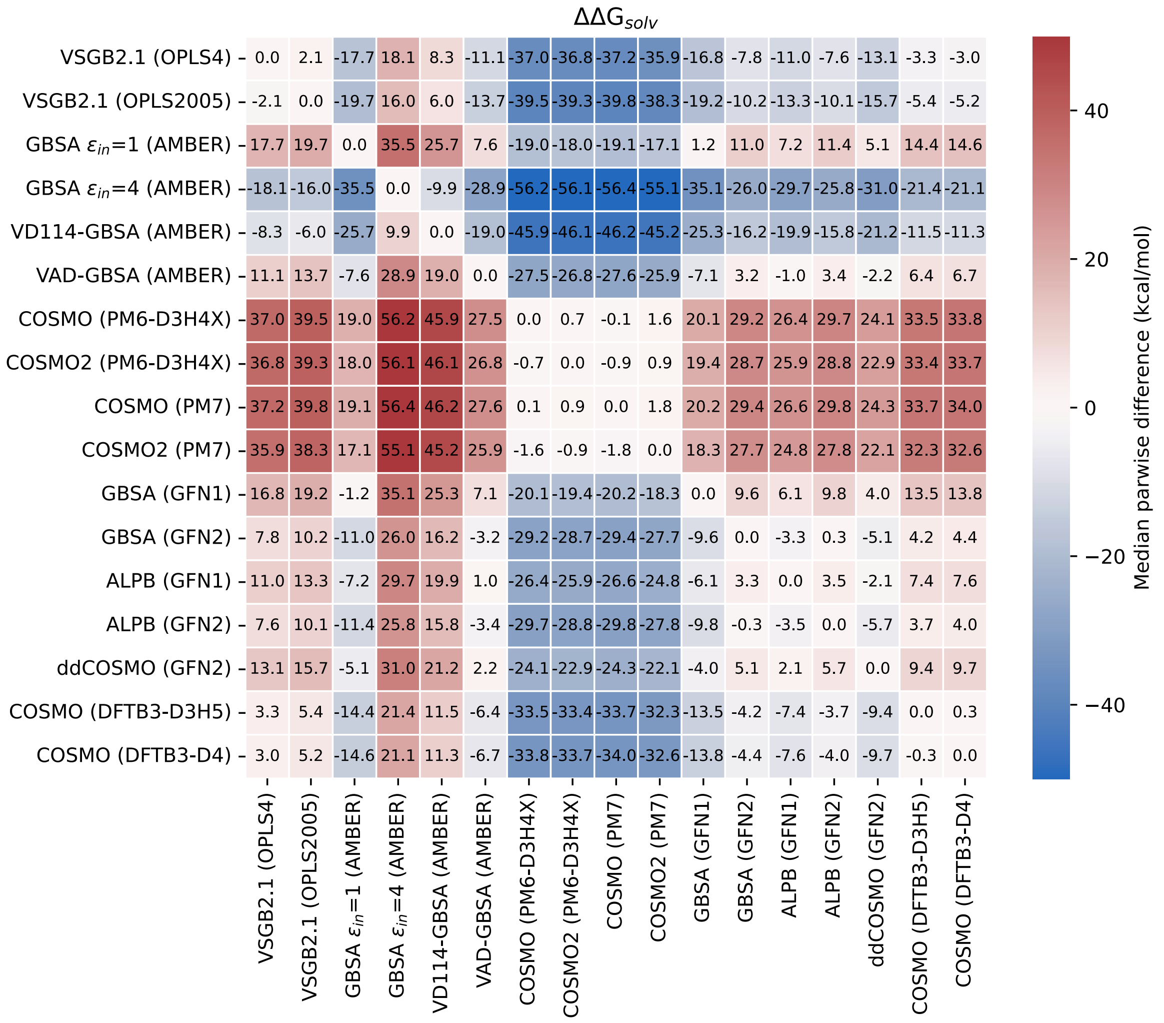

Figure S7. The same as S5, but for $\Delta\Delta G_{solv}$ term.

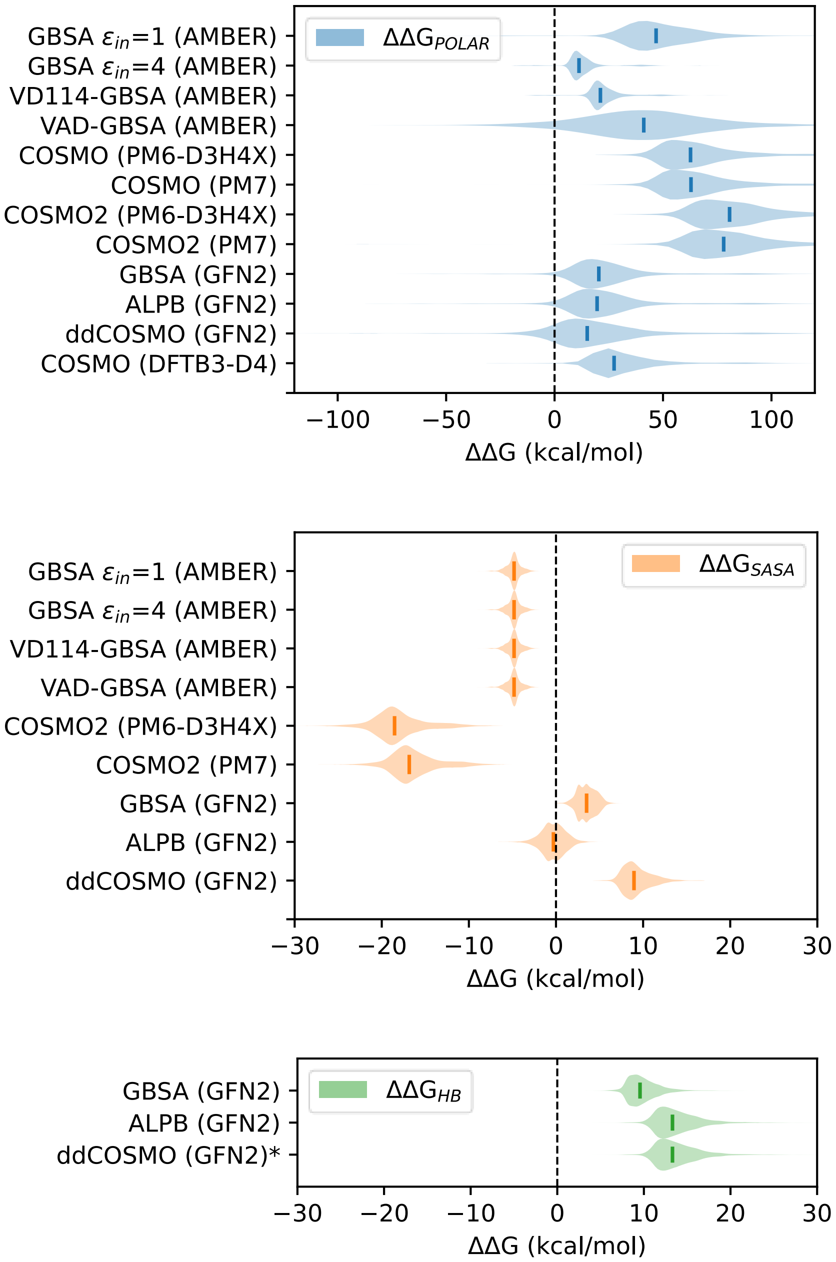

Figure S8. Comparison of the solvation contributions $\Delta\Delta G_{POLAR}$, $\Delta\Delta G_{SASA}$ and $\Delta\Delta G_{HB}$ as computed by different solvation models. Solid lines inside the violins indicate median values. All 10849 poses are included in the analysis.
